# Sodium and potassium urinary excretion and their ratio in the elderly: results from the Nutrition UP 65 study

**DOI:** 10.29219/fnr.v62.1288

**Published:** 2018-02-27

**Authors:** Pedro Moreira, Ana S. Sousa, Rita S. Guerra, Alejandro Santos, Nuno Borges, Cláudia Afonso, Teresa F. Amaral, Patrícia Padrão

**Affiliations:** 1 Faculdade de Ciências da Nutrição e Alimentação, Universidade do Porto, Porto, Portugal; 2 EPIUnit – Instituto de Saúde Pública, Universidade do Porto, Porto, Portugal; 3 Centro de Atividade Física, Saúde e Lazer, Universidade do Porto, Porto, Portugal; 4 Escola Superior de Saúde, Instituto Politécnico de Leiria, Leiria, Portugal; 5 I3S-Instituto de Investigação e Inovação em Saúde, Porto, Portugal; 6 CINTESIS–Centre for Health Technology and Services Research, Porto, Portugal; 7 UISPA-IDMEC, Faculdade de Engenharia, Universidade do Porto, Porto, Portugal

**Keywords:** Sodium, potassium, sodium-to-potassium ratio, elderly, urinary excretion

## Abstract

**Background:**

We aimed to describe urinary sodium and potassium excretion and their ratio in a representative sample of Portuguese elderly population, according to sociodemographic characteristics and weight status.

**Methods:**

A cluster sampling approach was used, representing older Portuguese adults (≥65 years) according to age, sex, education level, and regional area within the Nutrition UP 65 study. This cross-sectional evaluation was conducted in 2015 and 2016. From a sample size of 1,500 participants, 1,318 were eligible for the present analysis, 57.3% were women, and 23.5% were aged ≥80 years. Sodium and potassium consumption was evaluated through one 24 h urinary excretion. Inadequate sodium intake was defined as ≥2,000 mg/day, inadequate potassium intake was considered as <3,510 mg/day, and inadequate sodium-to-potassium ratio was defined as >1, according to the World Health Organization cutoffs.

**Results:**

The proportion of the participants with an inadequate intake was 80.0% in women and 91.5% in men (sodium), 96.2% of women and 79.4% of men (potassium), and 98.4% of women and 99.1% of men (sodium-to-potassium ratio). Higher sodium adequacy was observed among the older elderly, unmarried, with lower household income, and underweight/normal weight. Higher potassium adequacy was observed in the younger elderly, married, and with higher income.

**Conclusion:**

The majority of the Portuguese elderly population was classified as having inadequate sodium, potassium, and sodium-to-potassium ratio urinary excretion. Therefore, strategies for reducing sodium and increasing potassium intake are priorities in the Portuguese elderly population.

Over the past 25 years, the estimated deaths attributed to high blood pressure have increased considerably worldwide (1). In Portugal, despite improvements in the treatment of hypertension (2) and the decrease in the mean systolic blood pressure in adults, including elders, between 1990 and 2015 (1), cerebrovascular diseases are still the leading causes of death (2).

Jointly, cardiovascular diseases (CVDs) and cancer are responsible for 54% of all deaths (3). These two groups of diseases also share some important risk factors (4), and high sodium intake is recognized to increase the risk of fatal stroke, fatal coronary heart disease (5), cancer of the nasopharynx and stomach (6), and type 2 diabetes (7).

Potassium is also a critical mineral when assessing the health effects of sodium consumption, considering that this nutrient may mitigate the negative effects of excessive sodium intake by reducing blood pressure and thus preventing stroke (8, 9).

Additionally, the ratio of Na/K may be more reliable to assess the risk of CVD and CVD-related mortality than either sodium or potassium intake alone (10). However, studies on the relations of Na/K ratio with stroke or cardiovascular-related mortality have been sparse (11). To decrease blood pressure and risk of CVD, stroke, and coronary heart disease in adults, the WHO recommends a reduction to less than 2,000 mg/day of sodium (5) and a potassium intake of at least 3,510 mg/day (12). If individuals consume the amounts of sodium and potassium recommended by the WHO, the molar Na/K ratio will be approximately one to one, a ratio that is considered positive for health (5, 12). In this sense, lowering the Na/K ratio may reduce CVD risk and mortality (11), particularly in the elderly (13), although studies on the optimal relation between Na and K intake are still scarce (11).

Current estimates of sodium and potassium intake in a survey conducted between 2011 and 2012 in a representative sample of adults (18–90 years) from the Portuguese continental area estimated a mean 24 h urinary sodium excretion of 4,197 mg/day (10.7 g of salt) (14). In this study (14), mean 24 h urinary potassium excretion was 2,828 mg/day, making the Na/K ratio for all samples 2.5. In this study, data on sodium and potassium intake was provided for the overall sample, not allowing the characterization of older subjects.

The health in Portugal has improved considerably in the last decades (15), and the average life expectancy in the Portuguese population was 80.6 years in 2015 (16). However, the lack of data on sodium, potassium, and Na/K ratio intake in the elderly remains a challenge for the Portuguese region. The objective of our study is to describe 24 h urinary sodium and potassium excretion and their ratio in a representative sample of Portuguese elders, according to sociodemographic characteristics and weight status. The present study is a subproject of the Nutrition UP 65 study, whose protocol is published elsewhere (17).

## Methods

### Study design and sampling

A cross-sectional observational study was conducted in Portugal in a sample of 1,500 older Portuguese adults, ≥65 years old. To achieve a nationally representative sample of older Portuguese adults, a quota sampling approach was adopted using data from Census 2011 regarding sex, age, educational level, and regional area (defined in the Nomenclature of Territorial Units for Statistical purposes – NUTS II).

The potential participants were contacted by the interviewers, who provided information about the study purpose and methodology and invited them to participate. Individuals presenting any condition that precluded the collection of urine, such as dementia or urinary incontinence, were excluded from the study.

Data were collected between December 2015 and June 2016.

### Ethics

This research was conducted according to the guidelines established by the Declaration of Helsinki and the study protocol was approved by the Ethics Committee of the Department of Social Sciences and Health (Ciências Sociais e Saúde) from the Faculdade de Medicina da Universidade do Porto (no. PCEDCSS – FMUP 15/2015) and by the Portuguese National Commission of Data Protection (no. 9427/2015). All participants, or two representatives per participant, were asked to read and sign a duplicated informed consent form.

### Data collection

Sociodemographic data and nutritional status were collected using a structured questionnaire. Eight previously trained registered nutritionists were responsible for the questionnaire’s application and also by the anthropometric data collection.

Sociodemographic data included information on sex, age, regional area, education, marital status, residence type, and household income. The regional areas used are defined in NUTS II: Alentejo, Algarve, Azores, Lisbon Metropolitan Area, Center, Madeira, and North (18). Educational level was determined by the number of completed school years, and the following categories were used: no formal education, 1–3, 4, 5–11, and ≥12 years of school. Marital status was categorized as single, married or in a common-law marriage, divorced, or widowed. Residence type was defined as home or institution. Self-reported data regarding the presence of chronic diseases was also collected.

Body mass index (BMI) was computed using the standard formula [body weight (kg) /standing height ^2^ (m)], and participants were classified according to WHO cutoff values.

Anthropometric measurements were collected following standard procedures (19). Standing height was obtained with a calibrated stadiometer (Seca 213) with 0.1 cm resolution. For participants with visible kyphosis or when it was impossible to measure standing height due to a participant’s paralysis or due to mobility or balance limitations, height was obtained indirectly from non-dominant hand length (in centimeters), measured with a calibrated caliper from Fervi Equipment (Fervi Equipment, Vignola, Italy) with 0.1 cm resolution (20). Body weight (in kilograms) was measured with a calibrated portable electronic scale (Seca 803) (SECA GmbH, Hamburg, Germany) with 0.1 kg resolution, with the participants wearing light clothes. When it was not possible to weigh a patient, for the same reasons that prevented standing height measurement, body weight was estimated from mid-upper arm and calf circumferences (21). Mid-upper arm and calf circumferences were measured with a metal tape measure from Lufkin (Lufkin W606 PM, Lufkin, Sparks, Maryland, USA) with 0.1 cm resolution.

The volume of urine in a 24 h period was collected for each participant. The interviewers gave the participants oral and written instructions on how to proceed for the collection and storage of the volume of 24 h urine. A 24 h urine container was also provided. A certified laboratory, Labco (Lisbon, Portugal), was responsible for urine sample collection and analysis. Urinary creatinine was measured by the Jaffe method. A urine sample was considered inadequate if the creatinine level was <0.4 g/24 h for women or <0.6 g/24 h for men (22) or if the volume collected was <500 ml (23).

Sodium intake was evaluated after converting 24 h urinary sodium excretion, and excessive sodium intake was defined as ≥2,000 mg/day, according to the World Health Organization cutoffs (5). Potassium intake was also evaluated by 24 h urinary potassium excretion and was considered low if <3,510 mg/day. Na/K ratio >1 was defined as inadequate.

### Statistical analysis

Categorical variables were reported as frequencies. According to the normality of variable distribution, evaluated through Kolmogorov–Smirnov test, results were described as median and interquartile range (IQR).

Monthly household income was summarized using the following cutoffs: <€500, €500–€999, and ≥€1,000. Of the included participants, 645 (48.9%) did not know or preferred not to declare their income and thus they were allocated in a separate category.

Sodium and potassium intake, Na/K ratio median value, and the frequency of patients presenting inadequacy were compared across age groups, education, marital status, residence type, household income, and also for BMI using Kruskal–Wallis or Mann–Whitney test for continuous variables and Pearson chi-squared or Fisher’s exact test for categorical variables.

Results were considered significant when *p* < 0.05. Statistical analyses were conducted using the Software Package for Social Sciences for Windows (version 23.0, 2012, IBM (SPSS, Inc, an IBM Company, Chicago, IL)).

## Results

From the 1,500 individuals recruited, 178 were excluded because their urine samples were inadequate. Four other participants were excluded due to the impossibility of either measuring or estimating weight. The final study sample was composed of 1,318 participants, median (IQR) age equal to 73 (10) years, age ranging between 65 and 94 years, 57.3% women. Based on self-reported data, 65.2% participants (*n* = 859) reported having high blood pressure. Moreover, almost all participants (97.5%) mentioned presenting at least one chronic disease or prolonged health issue; eight participants did not know or preferred not to respond.

Compared to the final sample, the excluded participants were older, median (IQR) = 79 (12) years *versus* median (IQR) = 73 (10) years (*p* < 0.001), had attained lower educational level (*p* = 0.007), were less likely to be married or in a common-law marriage (*p* < 0.001), and were more likely to live in an institution (*p* < 0.001) and to have lower household income or to not declare their household income (*p* < 0.001).

All the analyses performed were stratified by sex. Regarding potassium intake and Na/K analyses displayed for men, one participant presenting a value of 0 mg/24 h potassium was excluded.

Height was estimated from hand length for 25 participants and weight was estimated from mid-upper arm and calf circumferences for 12 participants.

The older Portuguese adults within the present sample presented a median (IQR) sodium excretion of 3,368 (1,848) mg/day [equivalent to a median (IQR) salt excretion of 8.42 (4.62) g/day], a median (IQR) potassium intake of 2,262 (1,131) mg/day, and a median (IQR) Na/K of 2.33 (1.07).

Concerning sodium, 80% of women and 91.5% of men presented excessive intake. Otherwise, only 3.8% of women and 20.6% of men had an adequate potassium intake. Also, 98.4% of women and 99.1% of men presented Na/K ≥ 1.

As displayed in Table 1, among women, sodium intake decreased with age. Women aged 80 or more years presented a lower frequency of inadequate sodium intake than women aged 65–79 years. Notwithstanding this, potassium intake decreased across the two age groups and almost all (99.5%) women aged 80 or more years presented inadequate potassium intake. Women with no formal education presented lower potassium intake than women who attended school. However, no significant associations were found between education and sodium intake. Women who were married or in a common-law marriage presented higher sodium intake than single, divorced, or widowed women and the same tendency was observed for potassium intake. Women living at home ingested more sodium than those institutionalized. However, concerning the two residence types, no significant differences were found regarding potassium intake. Concerning household income, the highest sodium intake was observed for women with ≥€1,000 and the lowest potassium intake was observed for women with ≤€499 and for those who did not declare the household income. Sodium intake increased across BMI categories, with obese women presenting a higher frequency of inadequacy than underweight/normal and overweight women. Regarding potassium intake, no significant differences were observed across BMI categories. Concerning Na/K, there were no significant differences among women for any of the studied characteristics, with an exception for residence type: institutionalized women presented lower Na/K than women living at home.

**Table 1:** Sodium intake, potassium intake, and sodium-to-potassium ratio (Na/K) in 755 older Portuguese women, ≥65 years old, participating in a crosssectional observational study

	Sodium intake, mg/day (*n* = 755)		Potassium intake, mg/day (*n* = 755)		Na/K (*n* = 755)	
			
Age, years^a^	Median (IQR)	Inadequate (≥2,000) *n* (%)	Median (IQR)	Inadequate (<3,500) *n* (%)	Median (IQR)	>1*n* (%)
65–79	3,008 (1,532)	459 (82.0)	2,106 (897)	532 (95.0)	2.38 (1.10)	550 (98.2)
≥80	2,552 (1,168)	145 (74.4)	1,755 (780)	194 (99.5)	2.31 (1.02)	193 (99.0)
* p*	n.s.	<0.05	<0.05	<0.05	n.s.	n.s.
Education, years						
0	2,572 (1,860)	91 (72.8)	1,755 (1092)	121 (96.5)	2.50 (1.12)	122 (97.6)
1–3	3,064 (1,780)	132 (78.1)	1,950 (819)	161 (95.3)	2.43 (1.23)	166 (98.2)
4	2,856 (1,464)	290 (82.4)	2,106 (780)	339 (96.3)	2.33 (1.03)	346 (98.3)
5–11	2,692 (1,168)	66 (83.5)	1,989 (780)	76 (96.2)	2.28 (0.91)	79 (100)
≥12	2,760 (924)	25 (83.3)	1,989 (721.5)	29 (96.7)	2.10 (1.30)	79 (100)
* p*	n.s.	n.s.	<0.05	n.s.	n.s.	n.s.
Marital status						
Single/divorced/widowed	2,712 (1,428)	359 (76.4)	1,930.5 (897)	454 (96.6)	2.35 (1.11)	461 (98.1)
Married/common-law marriage	3,088 (1,452)	245 (86.0)	2,145 (780)	272 (95.4)	2.44 (1.00)	282 (98.9)
* p*	<0.05	<0.05	<0.05	n.s.	n.s.	n.s.
Residence						
Home	2,856 (1,520)	582 (80.9)	2,028 (897)	690 (96.0)	2.40 (1.07)	707 (98.3)
Care home	2,200 (1,328)	22 (61.1)	1,794 (828.8)	36 (100)	1.99 (0.96)	36 (100)
* p*	<0.05	<0.05	n.s.	n.s.	<0.05	n.s.
Income, €						
≤499	2,876 (1580)	130 (78.8)	1,950 (1014)	163 (98.8)	2.40 (1.24)	161 (97.6)
500–999	2,936 (1388)	147 (86.5)	2,184 (711.8)	163 (95.9)	2.37 (1.10)	169 (99.4)
>1,000	3,040 (1,660)	65 (87.8)	2,086.5 (858)	69 (93.2)	2.38 (1.15)	72 (97.3)
Unknown/no response	2,656 (1,520)	262 (75.7)	1,950 (867.8)	331 (95.7)	2.34 (0.98)	341 (98.6)
* p*	<0.05	<0.05	<0.05	n.s.	n.s.	n.s.
BMI, kg/m^2^						
Underweight/normal	2,400 (1,492)	74 (72.5)	1,911 (750.8)	100 (98.0)	2.23 (1.13)	98 (96.1)
Overweight	2,748 (1,480)	242 (77.1)	2,028 (897)	305 (97.1)	2.28 (0.96)	308 (98.1)
Obese	2,996 (1,756)	288 (85.0)	2,028 (936)	321 (94.7)	2.49 (1.12)	337 (99.4)
* p*	<0.05	<0.05	n.s.	n.s.	n.s.	n.s.

Note: 2,000 mg sodium = 5 g salt (NaCl).

n.s. – non significant

BMI, body mass index; IQR, interquartile range.

a 65–79 years: *n* = 560; ≥80 years: *n* = 195.

The results for men are presented in Table 2. Men aged 80 years or more presented lower sodium intake and also lower potassium intake than men aged 65–79 years. No differences were observed concerning education level for sodium intake. However, similarly to women, men with no formal education presented lower potassium intake than those who attended school. Both sodium and potassium intakes were higher for married men or those in a common-law marriage than for those who were single, divorced, or widowed. Also, a lower proportion of men who were married or in a common-law marriage presented inadequate potassium intake. Institutionalized men presented lower sodium intake than those living at home, whereas no differences were found regarding potassium intake. Men in the lowest category of household income presented the lowest sodium intake and also the lowest potassium intake. Sodium intake increased across BMI categories but no differences were observed for potassium intake. Similarly to what was observed among women regarding Na/K, only residence type presented significant differences, with a lower Na/K for institutionalized men.

**Table 2:** Sodium intake, potassium intake, and sodium-to-potassium ratio (Na/K) in 563 older Portuguese men, ≥65 years old, participating in a cross-sectional observational study

	Sodium intake, mg/day (*n* = 563)		Potassium intake, mg/day (*n* = 562)		Na/K (*n* = 562)	
			
Age, years^a^	Median (IQR)	Inadequate (≥2,000) *n* (%)	Median (IQR)	Inadequate (<3,500) *n* (%)	Median (IQR)	>1*n* (%)
65–79	3,812 (2,052)	416 (92.0)	2,730 (1,365)	351 (77.8)	2.31 (1.04)	448 (99.3)
≥80	3,484 (1,684)	99 (89.1)	2,496 (1,014)	95 (85.6)	2.24 (1.12)	109 (98.2)
* p*	<0.05	n.s.	<0.05	n.s.	n.s.	n.s.
Education, years						
0	3,416 (1,440)	44 (91.7)	2,340 (897)	43 (89.6)	2.44 (1.21)	48 (100)
1–3	3,774 (1,808)	69 (93.2)	2,691 (1,365)	58 (79.5)	2.32 (1.18)	73 (100)
4	3,860 (2,152)	284 (90.2)	2,769 (1,443)	241 (76.5)	2.25 (1.04)	310 (98.4)
5–11	3,768 (1,964)	90 (94.7)	2,730 (1,014)	77 (81.1)	2.29 (0.84)	95 (100)
≥12	3,628 (1,940)	28 (90.3)	2,379 (975)	27 (87.1)	2.13 (1.25)	91 (100)
* p*	n.s.	n.s.	<0.05	n.s.	n.s.	n.s.
Marital status						
Single/divorced/widowed	3,288 (1,668)	174 (88.8)	2,379 (1,014)	172 (88.2)	2.35 (1.14)	195 (100)
Married/common-law marriage	3,952 (2,064)	340 (92.9)	2,886 (1,326)	273 (74.6)	2.27 (1.02)	361 (98.6)
* p*	<0.05	n.s.	<0.05	<0.05	n.s.	n.s.
Residence						
Home	3,768 (1,952)	504 (91.8)	2,652 (1,248)	434 (79.2)	2.31 (1.05)	543 (99.1)
Care home	2,668 (1,320)	11 (78.6)	2,574 (1,267.5)	12 (85.7)	1.59 (0.90)	14 (100)
*p*	<0.05	n.s.	n.s.	n.s.	<0.05	n.s.
Household income, €						
≤499	3,276 (1,812)	46 (86.8)	2,418 (1,101.8)	46 (88.5)	2.36 (1.05)	50 (96.2)
500–999	3,908 (2,168)	109 (92.4)	2,691 (1,218.8)	96 (81.4)	2.39 (0.93)	118 (100)
>1,000	4,096 (1,584)	91 (97.8)	3,003 (1,287)	66 (71.0)	2.20 (0.98)	93 (100)
Unknown/no response	3,648 (2,036)	269 (90.0)	2,535 (1,287)	238 (79.6)	2.24 (1.12)	296 (99.0)
* p*	<0.05	n.s.	<0.05	n.s.	n.s.	n.s.
BMI, kg/m^2^						
Underweight/normal	3,324 (1,616)	89 (89.9)	2,457 (1,287)	84 (84.8)	2.18 (0.88)	93 (99.0)
Overweight	3,780 (1,932)	261 (91.9)	2,613 (1,326)	225 (79.5)	2.34 (1.16)	280 (98.9)
Obese	3,908 (2,188)	165 (91.7)	2,749.5 (1,238.3)	137 (76.1)	2.30 (0.97)	179 (99.4)
* p*	<0.05	n.s.	n.s.	n.s.	n.s.	n.s.

Note: 2,000 mg sodium = 5 g salt (NaCl).

BMI, body mass index; IQR, interquartile range.

n.s. – non significant

a 65–79 years: *n* = 451; ≥80 years: *n* = 112.

## Discussion

Our results have shown that above 64 years, 80% of the women and 91.5% of the men exceeded the current guideline on sodium consumption (5), while for potassium 96.2% of women and 79.48% of men did not meet the recommendation, and only 0.9–1.6% met the WHO reference Na/K ratio (12). Mean sodium and potassium intakes in men and women were well below the values reported for the general Portuguese adult population by the Physa Study (mean intakes for sodium were 4,268 mg in men and 4,094 mg in women and for potassium 2,980 mg in men and 2,940 mg in women) (16); however, these results are difficult to compare with our findings, since the results were not stratified by age in the latter study and subjects above 64 years of age represented only 23.3% of the total sample.

The present results reinforce the view that the vast majority of the world’s population have a sodium intake within the range of 2.5–5 g (24), which is far from the recommendation of no more than 2 g of sodium per day. However, it is worth mentioning that the current cutoffs regarding the recommended sodium and sodium-to-potassium ratio intakes are still controversial, and some authors (25) argue that a U-shaped curve describes the risk association of dietary sodium intake with CVD and all-cause mortality. Furthermore, a recent publication (26) assessed the associations of sodium intake with cardiovascular events, and this research is recognized as the largest individual-level data study relating sodium intake to CVD events and mortality; for those individuals without hypertension, compared with 4–5 g/day, higher sodium excretion was not associated with risk of the primary composite outcome (≥7 g/day), whereas an excretion of less than 3 g/day was associated with a significantly increased risk (26). However, the methodology of the previous study has been criticized particularly for the use of a morning spot urine sample to estimate usual salt intake and for the use of Kawasaki formula to estimate salt intake in individuals (27).

In addition, the vast majority of elderly Portuguese participants who reported having high blood pressure also reported the use of antihypertensive drugs (91.3%). This fact may contribute to an explanation for the apparently high age of the Portuguese even in the presence of high sodium intake.

Moreover, the existence of unknown confounding factors that make the population resistant to the adverse effects of high sodium intake is also a possibility (28, 29), and some authors have provided new insights into confounding variables involved in the control of sodium homeostasis that should be considered in future studies aiming to address public health issues about recommended salt intake (30).

Notwithstanding this, the current recommendations, which are internationally accepted (5), indicate the reduction of salt intake at population level as a public health priority (27).

In WHO European member states with salt consumption assessed by 24 h urinary excretion, estimates ranged between 8.25 and 18 g/day, with no member states meeting recommended levels (31). In the global report of Powles et al. (32) describing national sodium intakes by urine collection in 187 countries, the mean values in 2010 were 3.95 g/day (10.6 g salt/day), with the intake being about 10% higher in men than in women, while the differences by age were minor. In this systematic analysis, sodium intakes were higher in Eastern Europe (>4.2 g/day) than in Central Europe (3.9–4.2 g/day), and in Western Europe intakes ranged from 3.4 to 3.8 g/day. No stratification for sodium and potassium intakes after age 65 was provided in these previous mentioned reports.

Despite the heterogeneity between different populations, in the vast majority of populations, sodium consumption is well above recommended levels, while potassium intake is far below the minimum reference intake. Consequently, as in our study, the Na/K molar ratio tends to be much higher than the WHO recommendation.

The intake of sodium and potassium was similar across education groups, but consumption was lower in the older group (80 years or more) in both genders, which may be related to the expected decline in energy intake in this age group, considering the reduction of energy requirements (resting metabolic rate, thermogenesis, and physical activity) with aging (33) and, consequently, the lower intake of these two micronutrients, since they may be positively correlated with overall energy intake (34, 35). Nevertheless, the above-mentioned factors acting together in these two nutrients may have contributed to maintaining the approximate values for the Na/K ratio in the two age groups (65–79 and ≥80 years).

Married subjects from both sexes exhibited the worst nutritional adequacy for sodium and potassium intake, although no significant differences in Na/K ratio was observed in relation to their non-married counterparts. Married people have been described as having a significant advantage in health (36). Conversely, the divorced, separated, and widowed may have compromised health (36) and higher mortality (36, 37), as well as poorer diet quality (38), consuming more industrial meals and fewer homemade foods (39). However, the higher intakes of sodium and lower intakes of potassium in married subjects, in the present study, may reflect unhealthy dietary choices influencing the intake of those nutrients. Although little is known about the relation between marital status and sodium and potassium intake in the elderly, Kutob et al. (40) reported a worse diet quality in married women in comparison to those who were divorced/separated, and this data may highlight current social changes of being married, divorced, separated, never married, or widowed that may impact long-held assumptions about marital status and nutrition-related health (41, 42). Single elders may be prone to adapt their food choices in order to satisfy their personal needs (42), possibly being nutritionally conscious, which may contribute to better adequacy of sodium and potassium.

We also found that institutionalized elderly participants of both sexes presented lower sodium intakes and Na/K ratios, which could be related to the variety of chronic conditions that may put them under health care needs (43), and concurrent lower salt intake than noninstitutionalized subjects.

Sodium and potassium intakes exhibited opposite directions according to income categories. Higher incomes were associated with higher frequency of sodium inadequacy and lower prevalence of potassium inadequacy. Higher-income persons are more likely to consume a healthy diet than lower income people, and the diet of high-income groups is reported to be higher in potassium (44), in line with our findings. Regarding sodium, income has been described as a variable that does not affect 24 h urinary sodium excretion (45), although in a recent systematic review and meta-analysis to assess socioeconomic determinants of sodium intake in adult populations of high-income countries (46), about two-thirds showed higher sodium intake in subjects with low socioeconomic conditions.

Dietary sodium is strongly related to energy intake (35) due to its inclusion in a wide variety of foods and food preparations (10), and dietary sodium is sometimes described as having a detrimental impact on overweight and obesity in the life cycle, including in adults (47–50), although no studies have specifically addressed this association in persons above 65 years old. In our study, the median sodium intake increased with increasing BMI categories in both sexes, and sodium inadequacy also increased in women with increasing BMI categories (from normal/underweight to obesity). Sodium intake is recognized as a factor that may increase the risk of obesity (51) as a consequence of increased thirst and fluid intake (52), namely of sugary drinks, or as a result of the consumption of energy-dense foods that are also rich in sodium (for example, cheese or chips) (53, 54), or even through a direct effect on obesity (47, 53). However, since sodium intake, similar to overweight and obesity, is strongly related to energy intake, energy consumption may act as a confounder in the association between sodium and body mass, and, considering the absence of data on energy intake in the present study, no further analysis was performed to assess the independent association of sodium intake with overweight/obesity adjusting for energy intake.

Considering the high levels of sodium and potassium inadequacy in the present study, one can expect that extraordinary efforts should be made to simultaneously decrease sodium and increase potassium intake, since these two nutrients may be positively correlated (34, 35). In addition, they can also be positively correlated with overall energy intake (34, 35), although this may not always happen (54). In the case of elderly persons with low energy requirements, meeting the sodium recommendation could be easier, whereas getting enough potassium may be a much more difficult task (55).

Population-based sodium reduction strategies are potentially cost-effective (56), and as a governmental food policy strategy ‘soft regulation’ approaches combining targeted industry agreements and public education are considered to be highly cost-effective worldwide (57). Among preventive measures, as new science has emerged, the basic messages to consumers about the health impact of a high sodium intake have evolved, focusing not only on children and adults but also on elderly people (12, 58).

Low potassium intake has been associated with hypertension and adverse cardiovascular and renal outcomes (9, 59), whereas there is high quality evidence regarding the role of an adequate potassium intake in decreasing the risk of stroke (9) and in reducing blood pressure in hypertensive subjects without adverse effect on blood lipid concentrations, catecholamine levels, or renal function in adults. Nevertheless, potassium excess can be harmful in patients with impaired potassium excretion (59), which may be the case of persons taking some drugs (for example, potassium-sparing diuretics) or having some medical conditions, such as renal disease, diabetes, or severe heart failure (60) A possible solution to achieve adequate intakes of sodium and potassium in the elderly could be the adoption of a dietary pattern based on low energy and high nutritionally dense foods, such as the DASH (Dietary Approaches to Stop Hypertension) diet (61) or the Mediterranean diet (62), adopting healthy cooking practices. However, economic constraints may also be a barrier, considering the higher economic costs of adopting low energy dense and high nutritionally dense food patterns (63), particularly in the case of a potassium-dense diet (64) or a Mediterranean eating style (65)

Some limitations have to be acknowledged. The Nutrition UP 65 study is a crosssectional study and therefore no causal association between sodium and potassium urinary excretion and the associated factors can be inferred. Another possible limitation of the present study is that the present sample, although it can be regarded as large, only comprises 0.075% of the older Portuguese population. Also, the study included a single 24 h urine collection, which may not represent the usual dietary intake of subjects. However, since the study include a large representative sample of the Portuguese elderly population, the impact of the latter limitation is minimized. To the best of our knowledge, this is the first study presenting nationwide results of 24 h urinary excretion of sodium and potassium and the respective Na/K ratio, specifically for the elderly population, which we consider to be the main strength of the present work.

At least eight in every ten elderly Portuguese participants did not meet the sodium intake maximum recommendation, whereas nine out of ten participants did not meet the minimum potassium intake reference, and virtually all the elderly had an inadequate Na/K ratio. Therefore, reducing sodium and increasing potassium intake must be seen as key priorities in the Portuguese elderly population.

## Conflict of interest and funding

Nutrition UP 65 is funded by Iceland, Liechtenstein, and Norway through European Economic Area (EEA) Grants in 85% and by Faculdade de Ciências da Nutrição e Alimentação, Universidade do Porto in 15%. Iceland, Liechtenstein, and Norway sponsor initiatives and projects in various program areas, primarily focusing on reducing economic and social disparities. The European Economic Area Grants are managed by Administração Central do Sistema de Saúde through the Programa Iniciativas em Saúde Pública.
